# First-time anterior cruciate ligament injury in adolescent female elite athletes: a prospective cohort study to identify modifiable risk factors

**DOI:** 10.1007/s00167-021-06595-8

**Published:** 2021-05-07

**Authors:** M. K. Zebis, P. Aagaard, L. L. Andersen, P. Hölmich, M. B. Clausen, M. Brandt, R. S. Husted, H. B. Lauridsen, D. J. Curtis, J. Bencke

**Affiliations:** 1 Department of Physiotherapy, Faculty of Health, University College Copenhagen, Sigurdsgade 26, 2200 Copenhagen, Denmark; 2 Department of Sports Sciences and Clinical Biomechanics, Muscle Physiology and Biomechanics Research Unit, University of Southern Denmark, Odense, Denmark; 3 National Research Centre for the Working Environment, Copenhagen, Denmark; 4 Department of Health Science and Technology, Sport Sciences, Aalborg University, Aalborg, Denmark; 5 Department of Orthopaedic Surgery, Sports Orthopedic Research Center-Copenhagen (SORC-C), Copenhagen University Hospital, Amager-Hvidovre, Hvidovre, Denmark; 6 Physical Medicine & Rehabilitation Research – Copenhagen (PMR-C); Department of Physical and Occupational Therapy; Department of Clinical Research; Department of Orthopedic Surgery, Copenhagen University Hospital Amager-Hvidovre, Hvidovre, Denmark; 7 Department of Clinical Research, Copenhagen University Hospital, Amager-Hvidovre, Hvidovre, Denmark; 8 Team Danmark, The Elite Sport Organization of Denmark, Brondby, Denmark; 9 RUBRIC (Research Unit on Brain Injury Rehabilitation), Department of Neurorehabilitation, TBI Unit, Rigshospitalet, Denmark; 10 Human Movement Analysis Laboratory, Department of Orthopedic Surgery, Copenhagen University Hospital, Amager-Hvidovre, Hvidovre, Denmark

**Keywords:** Football, Handball, Female, Screening, ACL, Biomechanics, Electromyography

## Abstract

**Purpose:**

To identify modifiable biomechanical and neuromuscular anterior cruciate ligament (ACL) injury risk factors for first-time ACL injury in adolescent female elite football and team handball players.

**Methods:**

Adolescent female elite football and handball players with no previous ACL injury participated in the present study. At baseline, players were tested during side-cutting manoeuvres performed in a 3-dimensional motion analysis laboratory with concomitant electromyography (EMG) measurements. Maximal isometric lower limb muscle strength was assessed by handheld dynamometry. Players were prospectively followed for 2 years after baseline testing, and all magnetic resonance imaging (MRI) verified ACL injuries were registered. The effect of 16 risk factor candidates on the relative risk (RR) of ACL injury was estimated using Poisson regression analysis.

**Results:**

Ninety players (age 16.9 ± 1.2 years) were included in the analyses. Nine first-time ACL injuries (injury incidence 10.0% (95% confidence interval (CI) 5.4–18.6%)) were registered during the 2-year follow-up period. Four risk factor candidates were significantly associated with the risk of ACL injury: (1) hip flexion angle at initial contact (IC) [RR 0.56, 95% confidence interval (CI) 0.34–0.92], (2) internal knee rotation angle at IC [RR 1.13, 95% CI 1.08–1.19], (3) semitendinosus EMG activity 50 ms prior to IC [RR: 0.62, 95% CI 0.43–0.89], and (4) external hip rotator strength [RR: 0.77, 95% CI 0.66–0.89].

**Conclusion:**

Four distinct ACL injury risk factors related to the side-cutting manoeuvre were identified in a population of adolescent female elite football and team handball players with no previous ACL injury. As ACL injury typically occur during side-cutting, intervention programmes to modify these risk factors pose a promising strategy for ACL injury prevention in adolescent female elite football and team handball.

**Level of evidence:**

II.

## Introduction

A major concern in female cutting sports is the fact that female gender is associated with an increased risk of sustaining an anterior cruciate ligament (ACL) injury [26]. Notably, the ACL injury incidence is particularly high among adolescent female athletes [20, 28], with recent time trend analysis revealing that the incidence has continuously increased during the past 20 years [4].

One important step towards prevention is a better understanding of the complexity of ACL injury mechanisms. Accordingly, systematic video analyses of ACL injury sequences have revealed that across sports, one of the most hazardous sports-specific movements in relation to ACL injury is the side-cutting manoeuvre [e.g. 8, 15, 17]. Notably, such ACL injuries typically occur without any contact with other players (non-contact ACL injury) or no direct contact with the injured leg (indirect ACL injury) [35].

To identify biomechanical and neuromuscular ACL injury risk factors during high-risk sports movements, prospective study designs are needed. Nevertheless, only few prospective risk factor studies are found in the literature, and only a single study has measured concurrent 3D motion analysis and muscle activity (electromyography, EMG) [30], allowing for a combined analyses of biomechanical and neuromuscular risk factors. In that study [30], athletes were assessed during vertical drop jumping, which does not resemble the typical ACL injury situation (i.e. side-cutting) observed in sports like football and team handball [11, 16]. To date, only a single prospective study has evaluated lower limb muscle activity during a side-cutting manoeuvre as ACL injury risk factor in adult female elite football and team handball players [37]. Although adolescent female elite football and team handball players are at the highest risk of ACL injury [5], no prospective study has so far used concurrent 3D motion analysis and muscle activity recording to identify biomechanical and neuromuscular risk factors during a specific high-risk movement (i.e. side-cutting) in this athlete group. Thus, the aim of the present study was to identify biomechanical and neuromuscular ACL injury risk factors for sustaining first-time ACL injury during a side-cutting manoeuvre in adolescent female elite football and team handball players. Based on previous observations in the literature, it was hypothesised that the integrated biomechanical and neuromuscular analysis employed in the present study would enable to identify modifiable risk factors in a cohort of adolescent female elite football and team handball players.

## Materials and methods

### Subject sample, study design and data collection

The study was approved by the local Ethics Committee in the Capital of Denmark (H-2-2010-091). Eligible study participants were adolescent female football and team handball players performing at elite level and enrolled in one of the Danish National Team youth teams. Written information about the purpose and content of the study was sent out to all eligible participants and their parents, and all parents gave their written informed consent for their child to participate in the study in accordance with The Declaration of Helsinki. Participants were excluded if they were injured at the time of inclusion, precluding them from performing the test protocol, or had previously sustained an ACL injury.

Baseline side-cutting testing took place between January 2011 and December 2011, from which time point Magnetic Resonance Imaging (MRI) verified ACL tears were prospectively registered for a 2 year period, i.e. until December 2013. From the same cohort, the association of hamstring and quadriceps pre-activity between different ACL risk screening tests [11] and maximal lower limb muscle strength and muscle pre-activity during side-cutting [12] have been reported.

## Test procedures

### Side-cutting manoeuvre

All participants were tested during a side-cutting manoeuvre performed in a 3D motion capture laboratory (Copenhagen University Hospital, Amager-Hvidovre, Denmark). During laboratory testing, the subject started 5 m in front of an instrumented force plate and was instructed to perform the side-cutting manoeuvre as fast as possible to simulate an in-game situation. To best simulate a match situation, neither cutting angle nor run-in speed were standardised. The test was repeated until five approved trials were captured (i.e. trials in which the force plate was hit correctly).

### 3D biomechanical measurements

An 8-camera Vicon 612 system (Oxford, England) and an AMTI force plate embedded into the floor (MA, USA) were used to evaluate the side-cutting manoeuvre according to previously reported procedures [12, 38]. Because the risk of ACL injury is highest during the initial landing (within 0–40 ms after IC) [17], only the first 100 ms of the contact phase was used for the analysis of kinematic and kinetic parameters. The local maxima of the external joint moments during the first 100 ms in all three planes were obtained for each trial, and mean values of five trials for each subject were used for statistic evaluation [6]. Hip and knee joint angles at initial ground contact were obtained to describe the initial position of the lower limb during the side-cutting manoeuvre.

Based on the literature [10, 17] and previous work [6] evaluating the side-cutting manoeuvre, the following kinematic and kinetic parameters (candidate risk factors) were assessed during the contact phase of the side-cutting manoeuvre: knee flexion angle at IC (°), hip flexion angle at IC (°), knee adduction angle at IC (°), knee internal rotation angle at IC (°), knee flexion moment (Nm/kg BW), hip flexion moment (Nm/kg BW), knee adduction moment (Nm/kg BW) and hip internal rotation moment (Nm/ kg BW).

### EMG: assessment of neuromuscular activity

**Fig. 1:**
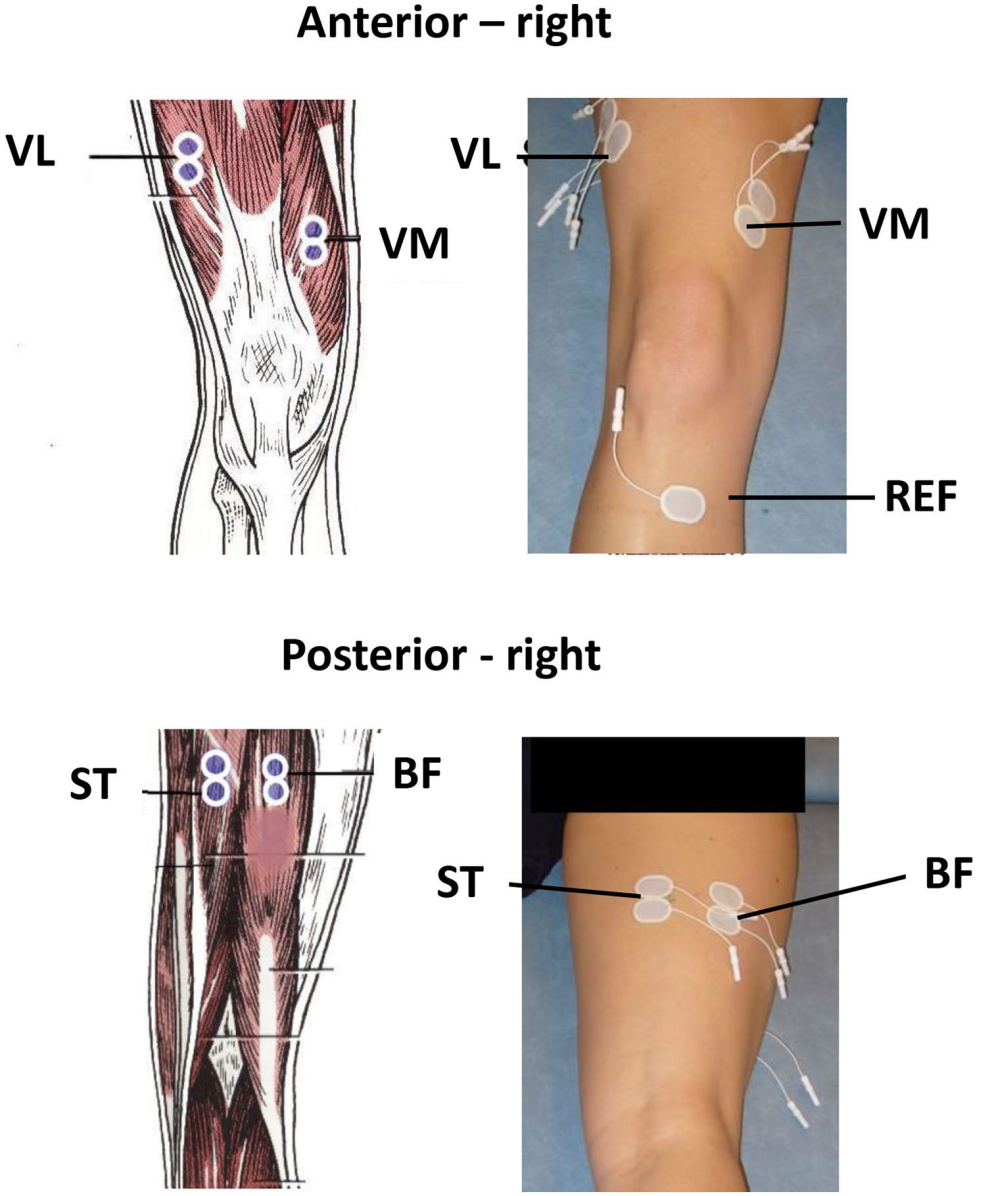
Surface electromyography (EMG) electrode placements of the quadriceps (anterior—right) and hamstring muscles (posterior—right). *VL* vastus lateralis, *VM* vastus medialis, *ST* semitendinosus, *BF* biceps femoris, *REF* reference electrode

During the side-cutting manoeuvre, neuromuscular activity was recorded (1000 Hz A/D conversion rate) in the quadriceps femoris muscle (vastus lateralis and vastus medialis) and hamstring muscles (semitendinosus and biceps femoris) of the preferred push-off leg using bipolar surface EMG electrodes (MyoMonitor IV, Delsys, Boston, MA, USA) [37] (Fig. 1). The raw EMG signals were recorded, highpass/lowpass filtered and normalised as described previously [12, 38].

**Fig. 2:**
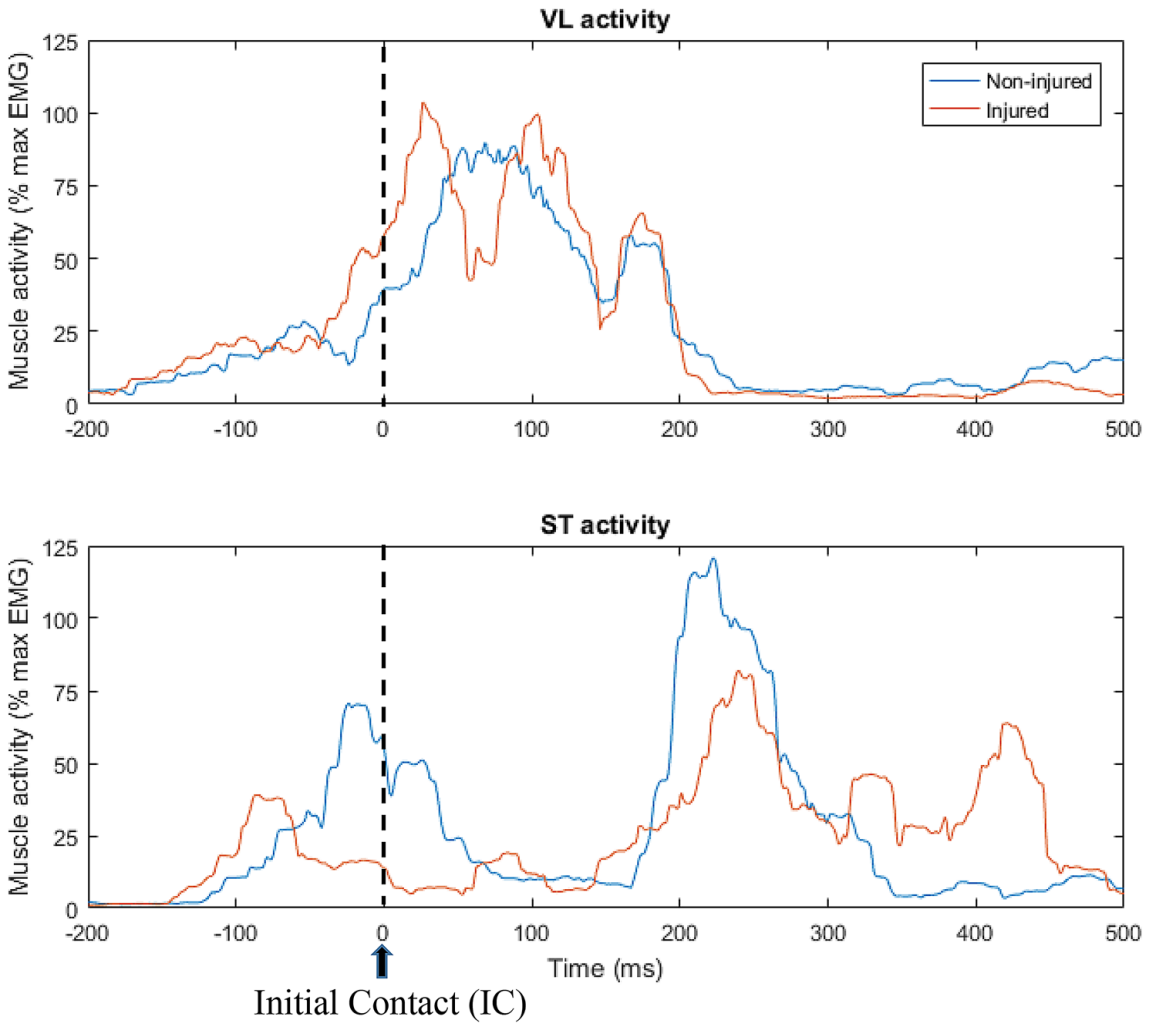
Muscle activity (% of max EMG) recorded during side-cutting. *VL* vastus lateralis, *ST* semitendinosus. Red line represents a player who subsequently sustained an ACL injury. Blue line represents a player who did not sustain an ACL injury in the study period. The black dotted line represents initial contact (IC)

The mean smoothed and normalised EMG amplitude was calculated for each muscle in the 50 ms time interval prior to IC registered by the force plate (Fig. 2). The average of five side-cutting trials was calculated for each player using synchronous force plate and EMG recording to quantify the magnitude and timing of muscle activity. The above neuromuscular and biomechanical variables obtained during the standardised side-cutting manoeuvre have previously demonstrated good-to-excellent within-session reliability [22, 39].

Based on previous reports evaluating the side-cutting manoeuvre [7, 37], the magnitude of pre-activity (50 ms before initial contact) in the examined muscles (i.e. VL, VM, ST BF) were included as potential ACL risk factors in the present analysis.

### Maximal isometric muscle strength

Maximal isometric muscle strength was measured with a portable hand-held dynamometer (PowerTrack II Commander, JTECH Medical, Salt Lake City, UT, USA) for the preferred push-off leg, as described by Askling et al. [3]. In each trial, the participant had four seconds to reach MVC. Participant performed three MVC trials for each muscle group, separated by 30 s of rest to avoid fatigue, and received strong verbal encouragement. The highest value for each muscle was used for analysis. Maximal hip and knee muscle forces were normalised to body weight (N/kg BW). The procedures for measuring maximal isometric knee flexor strength and maximal isometric hip abduction strength have been described in details elsewhere [12] and are characterised by a high test–retest reliability (ICC 0.84–0.98) [3, 32].

Maximal isometric external hip rotation strength was measured with the participant seated on an examination couch with both hips and knees in 90° of flexion. The participant was instructed to stabilise herself by holding onto the sides of the couch. The dynamometer was placed 5 cm proximal to the medial ankle malleolus. Measurements of maximal hip strength were performed in accordance with standardised procedures previously demonstrated to produce high degrees of reliability and no systematic test–retest bias [32, 33].

Maximal isometric knee extensor strength was not obtained since the dynamometer had an upper limit of 550 N. The peak EMG amplitude for vastus lateralis and medialis, respectively, was measured with the participant seated on an examination table and the leg statically fixed with an external strap at 60° knee flexion.

## Injury registration

All ACL injuries among the tested players using phone calls during the study period (until December 2013) were recorded. Detailed medical data and a description of the injury situation were obtained from all players reporting an ACL injury. The injury risk analysis included ACL injuries that were verified as total or partial rupture by magnetic resonance imaging (MRI), and categorised as a non-contact injury (i.e. occurring with no bodily contact with another player) or indirect contact injury (i.e. contact to any other body region other than the injured leg) [35]. Both categories were included in the present investigation, since video analyses have reported similar injury settings and lower limb kinematics in non-contact and indirect contact ACL injury situations [15, 35].

## Statistical analysis

The seasonal incidence of ACL injury and corresponding 95% confidence intervals (CIs) were estimated using Poisson regression analysis. The effect of each candidate risk factor on the relative risk (RR) of ACL injury (and corresponding 95% CIs) were also estimated using Poisson regression analyses, including the respective candidate risk factor as the dependent variable. RR estimates adjusted for sport (handball or football), were obtained through similar analyses, by including the variable sport as a covariate in the Poisson regression model. In order to improve the interpretability of the RR estimates, these were expressed in relation to clinically meaningful units [6, 37, 38] as listed in Table 1.

**Table 1:** The relative risk estimates (RR) in relation to clinically meaningful units

	Per 0.1 units increase	Per 1 unit increase	Per 10 units increase
Kinematics			
Knee flexion angle at IC (°)			X
Hip flexion angle at IC (°)			X
Knee adduction angle at IC (°)		X	
Knee internal rotation angle at IC (°)		X	
Kinetics			
Knee flexion moment (Nm/kg BW)	X		
Hip flexion moment (Nm/kg BW)	X		
Knee adduction moment (Nm/kg BW)	X		
Hip internal rotation moment (Nm/kg BW)	X		
Isometric muscle strength			
Knee flexion MVC (N/kg BW)	X		
Hip extension MVC (N/kg BW)	X		
Hip abduction MVC (N/kg BW)	X		
Hip external rotation MVC (N/kg BW)	X		
EMG pre-activity			
Vastus lateralis (% of max EMG)			X
Vastus medialis (% of max EMG)			X
Biceps femoris (% of max EMG)			X
Semitendinosus (% of max EMG)			X

*IC* initial contact, *MVC* maximal isometric voluntary contraction, *N* Newton, *BW* body weight, *EMG* electromyography

All Poisson regression analyses were performed using IBM SPSS v 25, with a 5% significance level.

## Results

### Study flow and participant characteristics

**Fig. 3:**
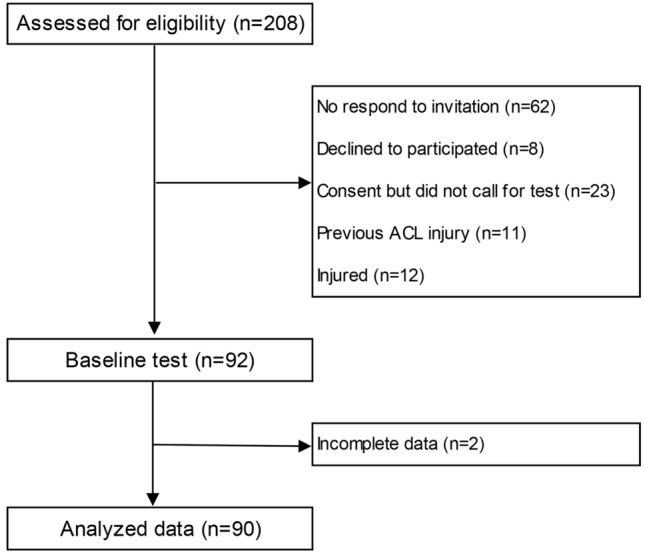
Flow diagram of included/excluded players. *ACL* anterior cruciate ligament

Two hundred and eight female athletes were assessed for eligibility. Ninety-two players, representing 33 different football and team handball clubs, were included in this prospective study of ACL injury risk in adolescent female football and team handball. After baseline testing, two players were excluded due to incomplete data collection. In total, 90 adolescent female elite football (*n* = 36) and team handball (*n* = 54) players (age 16.9 ± 1.2 years; height 172 ± 7 cm; weight 66 ± 8 kg) with 10 ± 2 years of experience with their sport were recruited for the study. An overview of participant flow is presented in Fig. 3. Baseline characteristics for the participants are reported in Table 2.

**Table 2:** Baseline characteristics of all participants (*n* = 90), uninjured players (*n* = 81) and subsequently ACL injured players (*n* = 9)

	Overall (*n* = 90)	Uninjured (*n* = 81)	ACL injured (*n* = 9)
Demographics			
Age, years	16.9 (1.2)	16.9 (1.2)	16.7 (1.3)
Height, cm	172 (7)	172 (7)	176 (5)
Weight, kg	66 (8)	66 (8)	69 (8)
Body mass index (kg/m^2^)	22 (2)	22 (2)	22 (2)
Sport (handball:football)	54:36	48:33	6:3
Experience (years)	10 (2)	10 (2)	9 (2)
Kinematics			
Knee flexion angle at IC (°)	25 (8)	26 (8)	21 (9)
Hip flexion angle at IC (°)	47 (10)	48 (10)	43 (6)
Knee adduction angle at IC (°)	1 (3)	1 (3)	1 (3)
Knee internal rotation angle at IC (°)	− 3 (6)	− 3 (5)	4 (10)
Kinetics			
Knee flexion moment (Nm/kg BW)	3.1 (0.5)	3.1 (0.5)	3.1 (0.5)
Hip flexion moment (Nm/kg BW)	3.8 (1.1)	3.8 (1.1)	3.9 (1.3)
Knee adduction moment (Nm/kg BW)	− 0.7 (0.5)	− 0.7 (0.4)	− 1.0 (0.7)
Hip internal rotation moment (Nm/kg BW)	0.3 (0.2)	0.3 (0.2)	0.4 (0.2)
Isometric muscle strength			
Knee flexion MVC (N/kg BW)	4.0 (0.6)	4.0 (0.6)	4.0 (0.6)
Hip extension MVC (N/kg BW)	3.7 (0.5)	3.7 (0.6)	3.8 (0.4)
Hip abduction MVC (N/kg BW)	2.6 (0.4)	2.6 (0.4)	2.4 (0.3)
Hip external rotation MVC (N/kg BW)	2.2 (0.3)	2.3 (0.3)	2.0 (0.1)
EMG pre-activity			
Vastus lateralis (% of max EMG)	33 (21)	34 (21)	27 (16)
Vastus medialis (% of max EMG)	38 (24)	39 (24)	28 (17)
Biceps femoris (% of max EMG)	32 (17)	33 (17)	24 (16)
Semitendinosus (% of max EMG)	46 (22)	48 (23)	32 (11)

Baseline characteristics are given as mean (SD)

*IC* initial contact, *MVC* maximal isometric voluntary contraction, *N* Newton, *BW* body weight, *EMG* electromyography

### Overall injury incidence

During the 2-year study period, nine players (six handball players, three football players) sustained an ACL injury on average 10 ± 6 months after baseline testing. Six of the nine ACL injuries were sustained during non-contact situations and three as indirect contact situations. Seven of the nine ACL injuries were sustained during match play. The overall injury incidence was 10.0% (95% CI 5.4–18.6%). Sport discipline (football vs. team handball) was not a risk factor in any of the analyses, Table 3.

**Table 3:** Risk factor candidates for ACL injury assessed during side-cutting (joint angles, joint moments, and EMG activation) and in lower limb strength testing (MVC)

	RR (95% CI)			
	Unadjusted	*P* value	Adjusted for sport	*P* value
Knee flexion angle at IC (°)^§^	0.47 (0.16–1.35)	0.159	0.43 (0.13–1.45)	n.s.
Hip flexion angle at IC (°)^§^	0.61 (0.39–0.99)	0.032	0.56 (0.34–0.92)	0.021
Knee adduction angle at IC (°)^#^	0.97 (0.76–1.23)	0.804	0.97 (0.77–1.24)	n.s.
Knee internal rotation angle at IC (°)^#^	1.12 (1.07–1.18)	< 0.001	1.13 (1.08–1.19)	< 0.001
Knee flexion moment (Nm/kg BW)*	1.01 (0.90–1.13)	0.878	1.00 (0.89–1.14)	n.s.
Hip flexion moment (Nm/kg BW)*	1.01 (0.96–1.07)	0.652	1.01 (0.96–1.07)	n.s.
Knee adduction moment (Nm/kg BW)*	0.90 (0.81–1.00)	0.050	0.90 (0.80–1.00)	n.s.
Hip internal rotation moment (Nm/kg BW)*	1.14 (0.77–1.69)	0.509	1.15 (0.77–1.73)	n.s.
Knee flexion MVC (N/kg BW)*	1.00 (0.89–1.12)	0.928	1.00 (0.88–1.13)	n.s.
Hip extension MVC (N/kg BW)*	1.03 (0.94–1.12)	0.558	1.04 (0.93–1.15)	n.s.
Hip abduction MVC (N/kg BW)*	0.89 (0.76–1.05)	0.161	0.89 (0.75–1.06)	n.s.
Hip external rotation MVC (N/kg BW)*	0.77 (0.68–0.89)	< 0.0001	0.77 (0.66–0.89)	0.001
Vastus lateralis (% of max EMG)^§^	0.84 (0.60–1.19)	0.332	0.85 (0.63–1.16)	n.s.
Vastus medialis (% of max EMG)^§^	0.79 (0.57–1.09)	0.149	0.79 (0.56–1.11)	n.s.
Biceps femoris (% of max EMG)^§^	0.68 (0.37–1.23)	0.196	0.67 (0.36–1.23)	n.s.
Semitendinosus (% of max EMG)^§^	0.65 (0.50–0.86)	0.002	0.62 (0.43–0.89)	0.010

Results from Poisson regression analyses

*IC* initial contact, *MVC* maximal isometric voluntary contraction, *N* Newton, *BW* body weight, *EMG* electromyography, *RR* relative risk, *CI* confidence interval, *n.s.* non-significant

*RR represents per 0.1 unit increase in outcome measure

^#^RR value represents per 1 increase in outcome measure

^§^RR value represents per 10 unit increase in outcome measure

### Kinematic risk factors

Hip flexion and internal knee rotation at initial contact was significantly associated with the risk of ACL injury, with a 44% decreased risk per 10° increase in hip flexion (RR 0.56 (95% CI 0.34–0.92), *P* = 0.021), and 13% increased risk per 1° increase in internal knee rotation (RR 1.13 (95% CI 1.08–1.19), *P* < 0.0001), Table 3.

### Kinetic risk factors

No relationships were found between any of the examined kinetic factors and ACL injury risk, Table 3.

### Muscle strength risk factors

As the only muscle strength parameter, maximal external hip rotator strength was associated with ACL injury risk. Thus, for every additional 0.1 N/kg BW increase in maximal external hip rotator strength, ACL injury risk decreased by 23% (RR 0.77 (0.66–0.89), *P* = 0.001), Table 3.

### Neuromuscular (EMG) risk factors

Reduced ST pre-activity was the only neuromuscular risk factor disposing for ACL injury. Specifically, the risk of ACL injury decreased by 38% when ST pre-activity increased by 10%-points during side-cutting (RR 0.62 (95% CI 0.43–0.89), *P* = 0.01), Table 3.

## Discussion

As the main finding of the present study, integrated biomechanical and neuromuscular screening allowed to identify four distinct and modifiable risk factors associated with increased risk of sustaining first-time ACL injury: (i) lower maximal hip external rotation strength, as well as (ii) more pronounced internal knee rotation at IC, (iii) decreased hip flexion at IC and (iv) reduced semitendinosus muscle pre-activity during side-cutting.

### Risk factor: maximal hip external rotation strength

Lower-extremity muscle strength is an important and modifiable factor for athletic performance and reduced lower-extremity muscle strength has been found to predict traumatic knee injury in young female athletes [27]. During the side-cutting manoeuvre, the hip extensors, hip external rotators, and hip adductors are the most loaded hip muscle groups during the critical early part of the stance phase [6]. In the present investigation, reduced external hip rotator strength was identified as a distinct ACL injury risk factor. In support of this observation, Khayambashi et al. reported low hip external rotator strength to be an independent predictor of non-contact ACL injury in a large-scale prospective study of male and female competitive athletes [14]. Reduced hip external rotator strength will reduce the capacity for producing compensatory joint moments to eliminate or minimise excessive internal hip rotation, which in closed kinetic chain conditions is known to force the knee joint into valgus positions, thereby imposing stress loads on the ACL [5]. The association between low external hip rotator strength and elevated risk of ACL injury observed in the present study strongly underlines the potential importance of increasing external hip rotator strength for the prevention of ACL injury. Nonetheless, previous ACL injury prevention programmes have not comprised specific strengthening exercises for the external hip rotators [1, 34]. As a novel recommendation, therefore, we propose that strength exercises for the hip external rotators should be included in future ACL injury prevention programmes.

### Risk factor: internal knee rotation at IC during side-cutting

In the transverse plane, increased internal knee rotation during side-cutting was associated with an increased risk of ACL injury. Biomechanically, internal rotation of the tibia results in increased loading of the ACL [9, 21]. In support of this notion, video analyses of ACL injury incidents suggest that internal tibia rotation in the time interval of 0–40 ms after IC is associated with ACL injury [15]. A recent meta-analysis examined if exercises used in injury prevention programmes modified cutting task biomechanics and found that none of the studies included could demonstrate changes in internal rotation at IC following training [25]. Thus, identifying exercises that target this particular risk factor during explosive-type movements (i.e. side-cutting) will have high clinical and practice relevance.

### Risk factor: hip flexion at IC during side-cutting

Individuals landing from jumps and performing cutting manoeuvres with extended hip and knee joint angles are exposed to elevated anterior tibial shear forces potentially resulting in increased strain in the ACL [29, 31]. The present data further demonstrate the importance of avoiding extended hip joint position during side-cutting. Revealing similar results, Leppänen et al. reported that for every 10° increase in hip flexion ROM (range of motion) during drop jump (DJ) landing, ACL injury risk decreased by 39% [19]. Performing side-cutting with extended hip joint position at the time of IC may create higher ground reaction impact forces that have to be controlled (and partly absorbed) by the muscles. In addition, high ground reaction impact forces will cause increased compression of the knee joint, which due to the posterior slope angle of the tibial plateau may induce internal tibial rotation, eventually leading to increased ACL strain [13]. Further, the functional importance of adopting more flexed hip joint angles during side-cutting manoeuvres may in part be that this position results in more elongated hamstring muscle lengths thereby optimising hamstring muscle force production due to the length-tension properties of this muscle group. None of the studies included in a recent meta-analysis have demonstrated changes in hip flexion angle at IC following training [25], highlighting the importance of identifying exercises that target this risk factor in injury prevention programmes.

### Risk factor: semitendinosus muscle pre-activity during side-cutting.

The present data supports previous findings in female adult elite football and team handball players, showing that reduced ST pre-activity during side-cutting predispose for ACL injury [37]. The side-cutting manoeuvre is a multi-planar movement, in which the medial and lateral hamstrings play different roles in controlling knee joint stability. The importance of medial hamstring pre-activity may be explained by the role of ST as a ‘knee adductor’, and serves an antagonistic role to the magnitude of external knee abduction moment by producing compression of the medial knee joint compartment [37]. Increased compression of the medial tibia-femoral compartment may further serve to countermeasure the magnitude of internal tibia rotation caused by the posteriorly increased slope of the lateral tibia plateau [13]. Although the present investigation did not find lateral hamstring muscle (BF) pre-activity to be associated with the risk of future ACL injury, it should be recognised that the BF muscle may still play an important role alongside ST in controlling anterior–posterior knee joint stability in the sagittal plane [13].

Interestingly, increased ST EMG muscle activation has been observed in female athletes during side-cutting after 6 weeks of agility training [36], and in response to 12 months multi-exercise intervention (balance/coordination, strength training, core stability and plyometric training) [39], respectively. More recently, this multi-exercise programme was combined with eccentric hamstring strengthening exercise (Nordic Hamstring), causing ST EMG activation to increase during side-cutting in adolescent female football and team handball players [38]. Collectively, these observations underline that considerable adaptive plasticity exist for this specific ACL injury factor. Thus, specific exercises that dominantly target the medial hamstring muscles may be identified to further optimise the prevention of ACL injury [40].

### Clinical implications

The present finding that adolescent female athletes with reduced preactivity of ST muscle during side-cutting are at increased risk of sustaining future ACL injury, suggests that ACL reconstruction using ST tendon autografts should be reconsidered. In support of this notion, a recent meta-analysis reported that the rate of recurrent ACL injuries was higher in patients operated with hamstring (ST) tendon autografts than bone-patella tendon-bone autografts and allografts [23]. Notably, high ACL re-rupture rates were reported in young female athletes [2]. Consequently, future studies comparing the effect of different ACL grafts on the risk of ACL re-injury explicitly in adolescent female athletes are warranted.

### Study strengths and limitations

Several potential limitations may be listed for the present investigation. First, the present high-risk movement (side-cutting) was evaluated in isolated laboratory conditions and not in a real-life context. However, in top level athletes this type of movement is the result of a consistent and highly reproducible motor programme [39], which makes it reasonable to expect that an identical host of risk factors will be present in a real-life context. Second, only the preferred (habitual) push-off leg and not the contra-lateral leg was examined. However, no side-to-side differences in external loading patterns seem to exist during the side-cutting manoeuvre [6].

Given the prolonged duration of the present study, it cannot be excluded that one or more risk factors may have changed during the time course of this study (e.g. with training or biological maturation). However, the present risk factor candidates have previously been found to remain consistently stable throughout long-term test–retest studies in young and adult female elite football and team handball players [10, 18, 39].

Methodological limitations may be observed with the use of EMG as a screening tool. Nevertheless, the present method of normalising all recorded EMG signal amplitudes to the peak EMG amplitude measured during MVC has previously been shown to result in highly reproducible EMG patterns when assessed during repeated trials of side-cutting [39].

Both elite football and team handball players were included in the present investigation since both athlete groups demonstrate a high risk of ACL injury, and because the side-cutting movement is recognised as a major risk factor in both sports [24, 35]. To account for potential differences between football and team handball in the biomechanics of the side-cutting movement, we included ‘sports discipline’ in the adjusted statistical analysis, which, however, did not alter the conclusion of the present study.

The experimental methods used in the present study to identify biomechanical and neuromuscular deficits are mostly applied to top level players only. Nonetheless, the present identification of distinct ACL injury risk factors can be used to optimise the choice of exercises used in the primary prevention performed by athletes at all competitive levels.

Finally, since the present study examined adolescent female elite football and team handball players, the present observations may not be generalizable to other age groups, participation levels or sport disciplines.

## Conclusions

Reduced external hip rotator strength as well as increased internal knee rotation angle, reduced hip flexion angle and reduced medial hamstring (ST) pre-activity during side-cutting were all identified as distinct risk factors disposing for elevated risk of first-time ACL injury. The present findings should be used to optimise the choice of exercises in order to target these risk factors in future ACL injury prevention programmes as well as in the late rehabilitation phase after ACL injury.
